# The Effects of Parenteral K1 Administration in Pseudoxanthoma Elasticum Patients Versus Controls. A Pilot Study

**DOI:** 10.3389/fmed.2018.00086

**Published:** 2018-04-16

**Authors:** Juan Luis Carrillo-Linares, María Inmaculada García-Fernández, María José Morillo, Purificación Sánchez, José Rioja, Francisco Javier Barón, María José Ariza, Dominic J. Harrington, David Card, Federica Boraldi, Daniela Quaglino, Pedro Valdivielso

**Affiliations:** ^1^Internal Medicine, Hospital Clínico Universitario Virgen de la Victoria, Málaga, Spain; ^2^Department of Human Physiology, University of Malaga, Málaga, Spain; ^3^Ophtalmology, Hospital Clínico Universitario Virgen de la Victoria, Málaga, Spain; ^4^Department of Medicine and Dermatology and Instituto de Biomedicina (IBIMA), University of Malaga, Málaga, Spain; ^5^Department of Preventive Medicine, Public Health and Science History, University of Málaga, Málaga, Spain; ^6^The Nutristasis Unit, Viapath, King’s Healthcare Partners, St. Thomas’ Hospital, London, United Kingdom; ^7^Department of Life Sciences, University of Modena and Reggio Emilia, Modena, Italy

**Keywords:** vitamin K, pseudoxanthoma elasticum, carboxylated proteins, osteocalcin, menaquinone

## Abstract

**Introduction:**

Pseudoxanthoma elasticum (PXE) is a rare disease caused by mutations in the ABCC6 gene. Vitamin K1 is involved in the posttranslational carboxylation of some proteins related to inhibition of the calcification process. Our aim was to investigate, in patients affected by PXE, baseline levels of vitamin K_1_-dependent proteins and -metabolites and whether parenteral administration of phytomenadione was effective in modulating their levels.

**Methods:**

We included eight PXE patients with typical clinical symptoms (skin, retina, and vascular calcification) and two ABCC6 causative mutations; 13 clinically unaffected first-degree patients’ relatives (9 carrying one ABCC6 mutation and 4 non-carriers). We assessed urinary vitamin K1 metabolites and serum Glu- and Gla-OC, Gas6 and undercaboxylated prothrombin (PIVKA-II), at baseline and after 1 and 6 weeks after a single intramuscular injection of 10 mg vitamin K1.

**Results:**

Comparison of PXE patients, heterozygous, and non-carriers revealed differences in baseline levels of serum MK-4 and of urinary vitamin K metabolites. The response to phytomenadione administration on vitamin K-dependent proteins was similar in all groups.

**Conclusion:**

The physiological axis between vitamin K_1_ and vitamin K-dependent proteins is preserved; however, differences in the concentration of vitamin K metabolites and of MK-4 suggest that vitamin K1 metabolism/catabolism could be altered in PXE patients.

## Introduction

Pseudoxanthoma elasticum (PXE) is a rare disease caused by mutations in the *ABCC6* gene encoding for the multidrug resistance-associated protein 6 (MRP6) highly expressed in the liver (1). The disease is characterized by progressive calcification of elastic fibers that undergo fragmentation and deformation with loss of function. Retinal damage at Bruch’s membrane causing central blindness, skin papules, and folds as well as vascular calcification responsible for ischemia are the most frequent clinical features (2).

The disease pathogenesis has not yet completely understood, though several hypotheses have been postulated. A series of *in vitro* and *in vivo* studies suggested that absent or non-functional MRP6 can influence the availability of circulating factors from the liver, which are physiologically required to prevent aberrant calcification and which are able to modulate the phenotype of mesenchymal cells, such as fibroblasts (3). As a consequence of fibroblasts’ abnormal protein profile (4), extracellular matrix components are differentially synthesized and/or degraded thus altering connective tissue homeostasis (4–6). Within this context, progressive mineralization of elastic fibers is probably sustained by an altered balance between factors inducing and inhibiting the mineralization process, such as fetuin A, Matrix Gla Protein (MGP), alkaline phosphatase, and pyrophosphate (7–11).

The association of vitamin K deficiency with ectopic calcification and with PXE-like phenotype (12, 13) has recently drawn attention to a number of proteins involved in ectopic calcification. Some of these proteins, such as MGP, osteocalcin (OC) and growth arrest-specific protein 6 (Gas6), require posttranslational vitamin K-dependent carboxylation to be fully active. The carboxylase enzyme uses the oxygenation of vitamin K hydroquinone to subtract a hydrogen from glutamyl residues (Glu) and to generate a carbanion that, incorporating CO_2_ by nucleophilic attack, produces a carboxylated-Glu known as Gla (14).

The observations that: (i) MGP levels were decreased in the serum of PXE patients compared to non-carriers (15), (ii) the concentration of MGP in the serum of Abcc6^−/−^ mice was similarly reduced when compared to wild-type animals and (iii) most of the MGP isolated from the liver of Abcc6^−/−^ mice was largely under-carboxylated, thus exhibiting reduced or no activity (16), suggested a possible pathogenic role of vitamin K in PXE (17). Consistently, serum vitamin K concentrations in PXE patients appeared significantly decreased compared to control subjects (18). Despite these findings, treatment of Abcc6^−/−^ mice with different forms of vitamin K failed to counteract the calcification process (19–21). In addition, *in vitro* experiments on fibroblasts isolated from controls and PXE patients demonstrated that these cells had a similar dose-dependent uptake of both vitamin K_1_ and K_2_ and had a significant increase of total protein carboxylation. By contrast, Gla-MGP was significantly lower in PXE fibroblasts (9), indicating that, in these patients, MGP cannot be efficiently carboxylated by fibroblasts, even in the presence of an adequate amount of the vitamin.

It remains questionable whether vitamin K metabolism is altered in PXE patients, since type and dosage of vitamin K have different effects depending on the model being studied (22, 23).

Therefore, we measured, for the first time in PXE patients and in unaffected subjects (i.e., heterozygous ABCC6 mutation carriers and non-carriers), baseline levels of vitamin K-dependent proteins and -metabolites and their changes after parenteral administration of a single dose of phytomenadione (vitamin K_1_).

## Patients and Methods

Pseudoxanthoma elasticum patients and unaffected individuals (patients’ first-degree relatives) were recruited through the Spanish Association of PXE Patients. Inclusion criteria for patients were: typical clinical features (i.e., skin and ocular involvement), skin biopsy positive to the von Kossa staining, presence of two ABCC6 causative mutations, either homozygous or compound heterozygous (24). Inclusion criteria for carriers were: complete absence of PXE clinical symptoms and one ABCC6 mutation. Inclusion criteria for non-carriers were: absence of PXE clinical manifestations and no ABCC6 mutations. In all cases, exclusion criteria were: negative informed consent, lack of molecular diagnosis, vegetarian diet, use of warfarin, intention to become pregnant during the study, age <18 years and hypersensitivity to vitamin K. Finally, eight PXE patients, nine heterozygous carriers, and four non-carriers were included in the study. All enrolled subjects were advised to keep to their usual diet for the duration of the study.

All participants in the study signed informed consent and the research was approved by the Ethical Review and Investigative Board of Hospital Universitario Virgen de la Victoria, Málaga, Spain.

Genetic analyses were carried out as previously described (25, 26).

EDTA, citrated and heparinized plasma, serum and urine samples were obtained at baseline and at 1 and 6 weeks after a single intramuscular injection in the buttock of 10 mg phytomenadione in 1 ml (Konakion©, Roche Pahrma, Madrid). Because subjects had similar body-mass index (Table 1), the same dose of 10 mg of phytomenadione was used for all subjects. Samples’ collection time points were selected in order to determine whether treatment has an effect on vitamin K-dependent proteins and if these effects are lasting. Effects of treatment may in fact be prolonged since vitamin K is extensively recycled prior to elimination, and supplementation is actually provided to individuals with normal nutritional intake and in the absence of any clinically relevant deficit of the vitamin (27).

**Table 1:** Baseline characteristics of pseudoxanthoma elasticum (PXE) patients, ABCC6 mutation carriers, and non-carriers.

	PXE patients (*n* = 8)	ABCC6 carriers (*n* = 9)	Non-carriers (*n* = 4)
Age (years)	39 ± 13	48 ± 17	57 ± 5
Sex (women)	6 (75%)	5 (55%)	1 (25%)
Weight (kg)	72 ± 11	65 ± 10	74 ± 4
BMI (kg/m^2^)	29 ± 5.1	25 ± 3	28 ± 4
Smoking			
Current	2 (25%)	4 (44%)	1 (25%)
Past	1 (12%)		
Alcohol	1 (12%)	2 (22%)	1 (25%)
Dyslipidaemia	2 (25%)	3 (33%)	1 (25%)
Systolic blood pressure (mmHg)	117 ± 12	110 ± 20	133 ± 10
Diastolic blood pressure (mmHg)	71 ± 18	68 ± 14	87 ± 8
Ankle–brachial index	1.12 ± 0.2	1.14 ± 0.08	1.25 ± 0.1
<0.9	1 (12%)	0	0
>1.4	2 (25%)	1 (10%)	1 (25%)

*Data are shown as mean ± SD or number (percentage)*.

Serum menaquinone-4 (MK-4) was measured by reversed-phase HPLC with fluorimetric detection (Waters 515). Chromatography was performed in isocratic conditions using an end-capped C18 column. Sample preparation was developed according to the protocol previously described (28).

Commercial ELISA kits were used to measure des-γ-carboxy prothrombin (DCP) (Prothrombin Induced by Vitamin K Absence or Antagonist II—PIVKA-II) in citrated plasma (Asserachrom, Diagnostica Stago, Asnière-sur-Seine, France), Fetuin A in EDTA-plasma (Quantikine, R&D System Inc., Abingdon, UK), Gas6 in serum (USCN, Life Science Inc., Houston, TX, USA), under-carboxylated (Glu) OC (EIA, Glu-Oc) in serum (Takara Bio Inc., Shiga, Japan), carboxylated (Gla) OC (EIA, Gla-OC) in heparinized plasma (Takara Bio Inc., Shiga, Japan).

Urinary 2-methyl-3-(5′-carboxy-3′-methyl-2′-pentenyl)-1,4-naphthoquinone (7C-aglycone) and 2-methyl-3-(3′-3′-carboxymethylpropyl)-1,4-naphthoquinone (5C-aglycone) metabolite derivatives of vitamin K were measured at each time point by HPLC with electromechanical detection, as previously described (29, 30). Urinary vitamin K metabolite concentrations were corrected for urinary creatinine concentration.

Statistical analyses were performed using “R: A Language and Environment for Statistical Computing.” Comparison between groups was performed using a linear mixed-model with PXE as fixed effect, adjusted by age and gender and the individual as a random effect along the repeated measurements.

## Results

Age, gender, and relevant clinical data are detailed for PXE patients, heterozygous carriers and non-carriers (Table 1). Standard laboratory parameters (hematology, coagulation parameters, glucose and lipid status, mineral and electrolytes, liver, kidney, and thyroid function) were similar in all subjects and vitamin K_1_ treatment did not significantly affect any of these parameters (data not shown).

Clinical manifestations of PXE involving skin, eyes, the vascular system, and other organs were described and quantified according to the Phenodex Score system (31). PXE patients with typical clinical manifestations always gave a total score ≥3 (Table 2). As expected, to all heterozygous carriers was attributed a 0 score since dermal, ocular, vascular, gastrointestinal, and cardiac symptoms were not observed (Table 3).

**Table 2:** Clinical and molecular features in pseudoxanthoma elasticum (PXE) patients.

Cases	Age	Mutations	Clinical manifestations					Phenodex Score	Other manifestations
			Skin (S)	Eye (E)	Vascular (V)	Gastrointestinal (G)	Cardiac (C)		
PXE 1	27	R1114C Q378X	Papules	Angioid streaks	None	None	None	Total score 3 S1/E2/V0/G0/C0	None

PXE 2	27	Del 23–29 Y62H	Laxity	Angioid streaks	None	bleeding	None	Total score 6 S3/E2/V0/G1/C0	None

PXE 3	33	Del 23–29 Y62H	Laxity	Angioid streaks	None	None	None	Total score 5 S3/E2/G0/V0/C0	None

PXE 4	33	R1141X R1141X	Laxity	Angioid streaks	None	None	None	Total score 5 S3/E2/V0/G0/C0	None

PXE 5	33	R1114C Q378X	Papules	Angioid streaks	None	None	None	Total score 3 S1/E2/V0/G0/C0	

PXE 6	43	R1141X R1141X	Laxity	Scarring	Intermittent claudication	None	Stroke	Total score 10 S3/E3/V2/G0/C2	Hypertension, kidney stones

PXE 7	57	R1114C Q378X	Laxity	Scarring	None	None	None	Total score 6 S3/E3/V0/G0/C0	Kidney stones

PXE 8	59	R600H R1314Q	Papules	Scarring	None	Bleeding	None	Total score 5 S1/E3/V0/G1/C0	Hypertension, kidney stones

**Table 3:** Clinical and molecular features in ABCC6 mutation carriers.

Cases	Age	Mutations	Clinical manifestations					Phenodex Score	Other manifestations
			Skin (S)	Eye (E)	Vascular (V)	Gastrointestinal (G)	Cardiac (C)		
C 1	23	R1314Q –	None	None	None	None	None	Total Score 0 S0/E0/V0/G0/C0	None

C 2	25	R1141X –	None	None	None	None	None	Total Score 0 S0/E0/V0/G0/C0	None

C 3	34	R1141X –	None	None	None	None	None	Total Score 0 S0/E0/V0/G0/C0	Hypertension, kidney stones

C 4	51	R600H –	None	None	None	None	None	Total Score 0 S0/E0/V0/G0/C0	None

C 5	52	R807Q –	None	None	None	None	None	Total Score 0 S0/E0/V0/G0/C0	Kidney stones

C 6	63	R600H –	None	None	None	None	None	Total Score 0 S0/E0/V0/G0/C0	None

C 7	65	R1141X –	None	None	None	None	None	Total Score 0 S0/E0/V0/G0/C0	None

C 8	67	R1141X –	None	None	None	None	None	Total Score 0 S0/E0/V0/G0/C0	Kidney stones

C 9	68	R1141X –	None	None	None	None	None	Total Score 0 S0/E0/V0/G0/C0	Atrial fibrillation

Table 4 show that MK-4 was higher in PXE patients at baseline compared to heterozygous and non-carriers. At 1 week post-vitamin K_1_ administration, MK-4 increased in all groups. Similar values were also observed at 6 weeks. Statistical analyses over repeated measurements indicate that, despite the similar behavior observed after treatment, differences between groups were maintained over the six week-period of the study (*p* < 0.05).

**Table 4:** Vitamin K metabolites and dependent proteins at baseline and at 1 and 6 weeks post-phytomenadione administration in pseudoxanthoma elasticum (PXE) patients, in non-carriers and in ABCC6 carriers.

Group	Baseline	Week 1	Week 6	Significance(along time)
**Vitamin K metabolites**				

**Serum MK-4 (ng/mL)**				
Non-carriers	15.5 ± 5.2	25.4 ± 12.4	23.7 ± 32.0	ns
ABCC6 carriers	20.0 ± 14.0	27.1 ± 12.4	23.1 ± 6.9	ns
PXE	28.8 ± 26.9	38.3 ± 32.0	41.6 ± 17.8	ns
Significance *(among groups)*	*^§^	*^§^	*^§^	
**Serum MK-7 (ng/mL)**				
Non-carriers	25.1 ± 8.8	22.8 ± 16.9	18.4 ± 7.8	ns
ABCC6 carriers	14.8 ± 11.3	23.4 ± 8.7	22.3 ± 8.0	ns
PXE	12.2 ± 8.7	23.0 ± 9.0	22.3 ± 2.0	ns
Significance *(among groups)*	ns	ns	ns	
**Urinary 5C-vitamin K metabolite (mg/g)**				
Non-carriers	6.86 ± 2.71	7.34 ± 2.82	4.21 ± 1.88	ns
ABCC6 carriers	3.87 ± 3.32	4.53 ± 3.53	3.15 ± 3.12	ns
PXE	3.27 ± 2.61	4.23 ± 4.35	3.63 ± 2.25	ns
Significance *(among groups)*	*	*	ns	
**Urinary 7C-vitamin K metabolite (mg/g)**				
Non-carriers	3.41 ± 1.48	3.98 ± 1.56	2.65 ± 1.22	ns
ABCC6 carriers	1.54 ± 1.16	2.55 ± 1.89	1.64 ± 0.90	ns
PXE	2.21 ± 1.33	2.49 ± 1.59	1.96 ± 1.28	ns
Significance *(among groups)*	ns	ns	ns	
**Total urinary vitamin K metabolites (mg/g)**				
Non-carriers	10.2 ± 4.19	11.3 ± 4.06	6.86 ± 2.89	ns
ABCC6 carriers	5.40 ± 4.45	7.07 ± 5.27	4.79 ± 3.80	ns
PXE	5.48 ± 3.71	6.72 ± 5.71	5.59 ± 3.26	ns
Significance *(among groups)*	ns	ns	ns	

**Vitamin K-dependent proteins**				

**Serum GAS6 (ng/mL)**				
Non-carriers	58.7 ± 7.61	52.8 ± 18.7	41.2 ± 5.4	*p* < 0.05
ABCC6 carriers	51.9 ± 9.18	46.3 ± 7.1	40.6 ± 7.4	*p* < 0.05
PXE	47.3 ± 9.14	47.9 ± 11.4	38.9 ± 8.6	*p* < 0.05
Significance *(among groups)*	ns	ns	ns	
**Serum Glu-Osteocalcin (ng/mL)**				
Non-carriers	7.0 ± 1.5	3.1 ± 1.5	5.9 ± 2.2	*p* < 0.05
ABCC6 carriers	14.6 ± 8.8	11.5 ± 8.9	13.9 ± 8.8	*p* < 0.05
PXE	11.7 ± 5.4	7.2 ± 2.6	10.1 ± 4.9	*p* < 0.05
Significance *(among groups)*	ns	ns	ns	
**Serum Gla-Osteocalcin (ng/mL)**				
Non-carriers	12.4 ± 5.5	18.6 ± 4.9	13.0 ± 6.0	*p* < 0.05
ABCC6 carriers	13.7 ± 5.9	20.2 ± 8.9	14.2 ± 5.4	*p* < 0.05
PXE	12.6 ± 8.3	15.9 ± 9.1	14.3 ± 9.6	*p* < 0.05
Significance *(among groups)*	ns	ns	ns	
**PIVKA-II (ng/ml)**				
Non-carriers	1.35 ± 0.56	1.13 ± 0.48	1.17 ± 0.72	ns
ABCC6 carriers	1.56 ± 0.58	1.29 ± 0.44	1.33 ± 0.41	ns
PXE	1.25 ± 0.38	1.13 ± 0.44	1.11 ± 0.50	ns
Significance *(among groups)*	ns	ns	ns	
**Serum Fetuin (ng/mL)**				
Non-carriers	893 ± 535	843 ± 498	925 ± 646	ns
ABCC6 carriers	959 ± 453	927 ± 364	813 ± 325	ns
PXE	795 ± 146	787 ± 227	834 ± 223	ns
Significance *(among groups)*	ns	ns	ns	

*p < 0.05 ABCC6 carriers or PXE versus non-carriers.

*^§^p < 0.05 ABCC6 carriers versus PXE*.

*Data shown as mean ± SD*.

**Figure 1:**
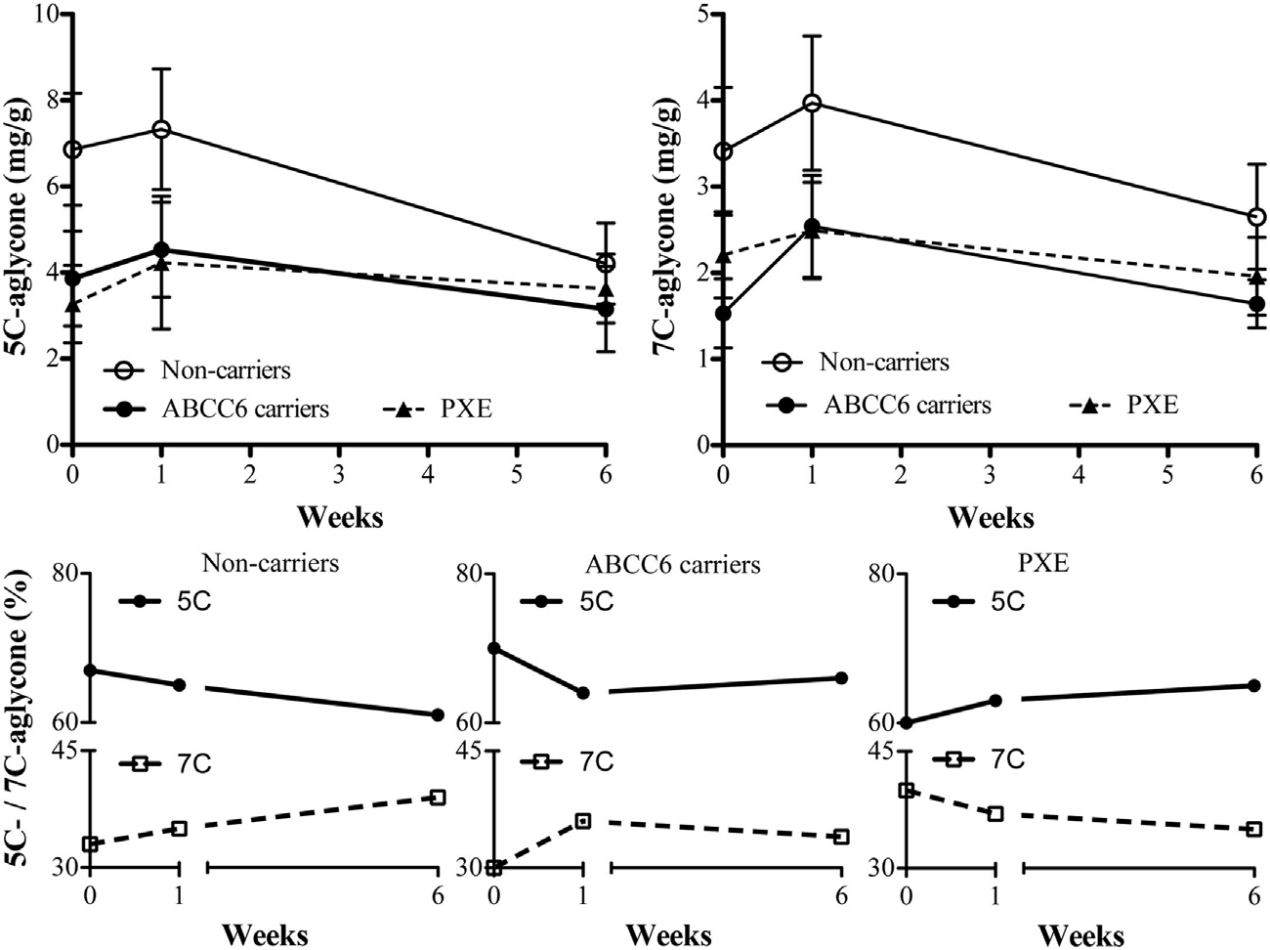
5C- and 7C-aglycone urinary vitamin K metabolites were measured by HPLC (mg/g creatinine) in pseudoxanthoma elasticum (PXE) patients, in heterozygous ABCC6 mutation carriers (ABCC6 carriers) and non-carriers at baseline (week 0) and after 1 and 6 weeks after parenteral phytomenadione administration. Data are expressed as mean values ± SD in upper panels and as percentage of 5C- or 7C-aglycone ratio on total aglycones in lower panels.

The concentrations of the 5C- and 7C-aglycone vitamin K metabolites were measured at baseline and after treatment, since their levels reflect total vitamin K status. Urinary volume excretion did not reveal significant differences between subjects (data not shown). As shown in Table 4, vitamin K_1_ was largely metabolized to terminal 5C-aglycone in all groups. Interestingly, 5C-aglycone levels in PXE patients and ABCC6 mutation carriers were significantly lower than those in non-carriers (*p* < 0.05). A similar trend was observed also as far as 7C-aglycone. To be noted that differences between groups were evident already at baseline. Figure 1 (lower panels) shows the percentage of 5C- or 7C-aglycone on total aglycones (5C + 7C) measured in non-carriers, ABCC6 carriers and PXE patients. These values allow to compare, within each group, how vitamin K_1_ was metabolized with time. In particular, in both non-carries and ABCC6 carriers, 7C-aglycone levels progressively increased over time, whereas an opposite trend was observed in PXE patients.

In order to investigate the effects of vitamin K_1_ supplementation, several vitamin K-dependent proteins were measured. Table 4 shows that PIVKA-II concentration were similar in PXE patients, in non-carriers and in ABCC6 carriers, at both baseline and post-vitamin K administration.

Serum Gas6 protein was similarly expressed at baseline in all groups (Table 4). Vitamin K_1_ administration diminished Gas6 concentration in all groups. Statistical analyses over repeated measurements revealed significant differences after treatment compared to baseline.

**Figure 2:**
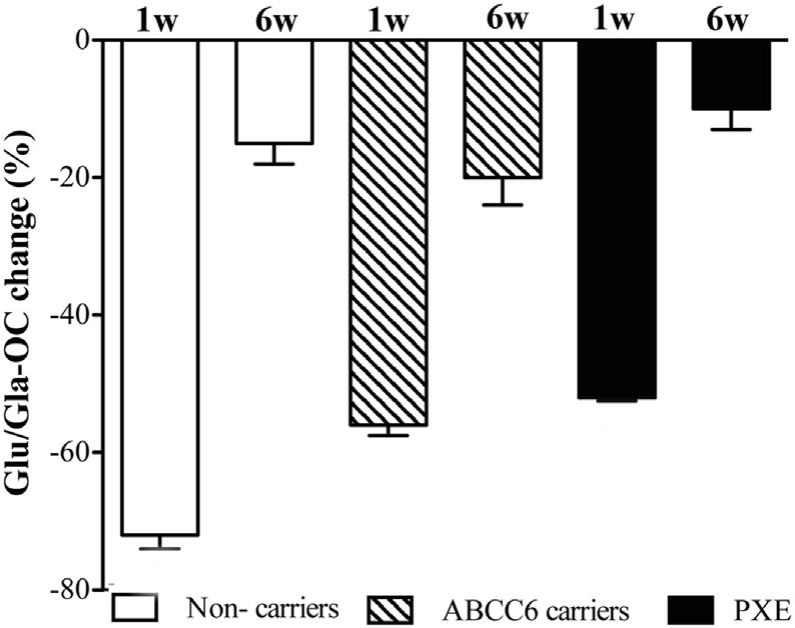
Ratio of circulating Glu-osteocalcin (Glu-OC) and Gla-osteocalcin (Gla-OC) measured by ELISA in pseudoxanthoma elasticum (PXE) patients, in heterozygous ABCC6 mutation carriers (ABCC6 carriers) and in non-carriers after 1 and 6 weeks post-phytomenadione administration. Data are expressed as percentage reduction (means value ± SD) compared to baseline (*p* < 0.05 along time for the three groups).

Figure 2 shows the effect of phytomenadione supplementation on the carboxylation status of OC, an extra-hepatic vitamin K-dependent protein involved in the calcification process. At baseline, both under-carboxylated (Glu)- and carboxylated (Gla)-OC were similar in all groups (data not shown). At 1 week post-treatment, the ratio between Glu- and Gla-OC was significantly reduced in all groups (*p* < 0.001). At 6 weeks after treatment, values returned to baseline concentrations (Figure 2).

Finally, as shown in Table 4, fetuin A levels, as expected for a non-vitamin K-dependent protein, did not change significantly over time.

## Discussion

It is well known that vitamin K has a role in the occurrence of ectopic calcification (32). Although suboptimal vitamin K status has been observed in PXE patients, thus suggesting that this vitamin could exert a key role in the defective carboxylation of MGP (8, 15, 16, 18); however, other experimental evidence does not support this hypothesis [10, 19, 21]. Nevertheless, it remains uncertain whether vitamin K metabolism/catabolism is altered in PXE patients.

The increased serum MK-4 concentration detected after phytomenadione administration indicates that the conversion of vitamin K_1_ to MK-4 is preserved in PXE, confirming previous *in vitro* data (9). However, differences at baseline and after treatment between patients and unaffected individuals may suggest possible altered regulation of metabolite production and/or in excretion pathways.

The significance of urinary aglycones measurements, as biomarkers of vitamin K status, resides in the observation that these metabolites reflect the excretion of total vitamins K, including MK-4 formed by conversion of vitamin K_1_ (29). In all subjects the excretion of the 5C-aglycone was greater than that of 7C-aglicone. However, at baseline, PXE patients exhibited reduced excretion of the urinary 5C-aglycone, indicating a suboptimal vitamin K status (18). Moreover, after vitamin K_1_ supplementation, non-carriers and ABCC6 carriers showed an increase of urinary 7C—aglycone excretion, whereas an opposite trend was present in PXE patients. Although the significance of these data could be explored also in the light of biliary excretion pathways (29), data suggest that vitamin K metabolism is modified in PXE.

To further investigate the effects of parenteral vitamin K_1_ supplementation, we measured the abundance of DCP, also known as prothrombin induced by vitamin K absence (PIVKA-II), an abnormal prothrombin molecule with diminished coagulation activity as a consequence of incomplete hepatic vitamin K-dependent carboxylation (33, 34). In this study, DCP levels, as well as coagulation test parameters, were similar in PXE patients and in heterozygous carriers and in non-carriers, confirming that vitamin K-dependent coagulation factors are not affected in PXE. By contrast, PXE-like manifestations due to mutations in the gamma-glutamyl carboxylase gene are associated to altered carboxylation of coagulations factors (18).

To evaluate the effects of phytomenadione administration on extra-hepatic tissues, we measured plasma levels of Gas6, a vitamin K-dependent protein that, differently to the homologous anticoagulant protein S (35, 36), is synthesized by fibroblasts, endothelium, vascular smooth muscle, and bone marrow cells (37, 38), although its role in the calcification process still remains on a speculative basis (39). According to our results, plasma levels of this protein, at baseline, were similar in all groups and progressively decreased with time, without significant difference between patients and unaffected subjects. Since vitamin K-dependent carboxylation of Gas6 favors protein binding to the Axl tyrosine kinase receptors (40), it could be suggested that the decrease in Gas6 levels observed in this study could be related to increased binding and consequently to reduced availability of free Gas6 in serum, further supporting the concept that vitamin-K dependent carboxylation *per se* is not affected in PXE.

To further assess the effects of parental phytomenadione administration, we have evaluated the carboxylated (gla) and undercarboxylated (glu) forms of OC, a protein involved in the remodeling of calcified matrix (14). In agreement with previous data (18) baseline glu-OC and gla-OC levels were not modified in PXE compared to unaffected subjects. Moreover, results support the observation that PXE patients similarly to unaffected subjects respond to vitamin K administration (10) by increasing carboxylated-OC. Since, vitamin K is believed to prevent vasculopathy (41), the hypothesis that prolonged elevation of vitamin K-dependent Gla-OC may delay tissue calcification could be explored in future studies.

Finally, the observation that fetuin A, a non-vitamin K-dependent α-glycoprotein synthesized by hepatocytes, is not affected by treatment, is a clear demonstration that changes observed after phytomenadione administration are the result of the treatment and not of non-specific metabolic interactions, e.g., acute phase response.

It has to be taken into account that PXE is a rare disorder. A restriction of the present investigation is related to the low number of patients enrolled and the resulting limited statistical power. Nevertheless, results from this pilot study indicate that, in PXE, phytomenadione might be able to positively influence the g-carboxylation process, thus indicating a proof of concept that vitamin K may actually exert some beneficial effects on calcification-related proteins.

## Conclusion

In conclusion and examining vitamin K-dependent proteins and vitamin K metabolites, this pilot study indicates that PXE patients demonstrate altered vitamin K metabolism compared to heterozygotes and non-carriers. Despite this, the response to parenteral phytomenadione administration is similar in patients and in unaffected individuals, suggesting that the physiological axis between vitamin K and vitamin K-dependent proteins is preserved in PXE. Because vitamin K is known to prevent vasculopathy (41), the elevation of Gla-OC seen in PXE patients after phytomenadione administration, may suggest that vitamin K treatment, if sustained over time, could contribute to delay tissue calcification.

## Ethics Statement

The present study was approved by the Comité de Ética de la Investigación Clínica de Málaga. All participants signed the informed consent.

## Author Contributions

JC participated in the design of the study, carried out patient’s data capture and helped to draft the manuscript. MG-F participated in the design and performed laboratory analyses. MM and PS were in charge of the patient’s management and participated in the data capture. JR carried out biochemical analysis. FJB performed the statistical analysis. MA participated in the genetic analysis. DH and DC carried out the vitamin K metabolites determination. FB and DQ coordinated the genetic analyses and helped to draft the manuscript. PV conceived of the study, participated in its design and coordination and drafted the manuscript. All authors read and approved the final manuscript.

## Conflict of Interest Statement

The authors declare that the research was conducted in the absence of any commercial or financial relationships that could be construed as a potential conflict of interest.
